# Experimental philosophical bioethics and normative inference

**DOI:** 10.1007/s11017-021-09546-z

**Published:** 2021-11-17

**Authors:** Brian D. Earp, Jonathan Lewis, Vilius Dranseika, Ivar R. Hannikainen

**Affiliations:** 1 Departments of Philosophy and Psychology, Yale University, New Haven, CT USA; 2 Uehiro Centre for Practical Ethics, University of Oxford, Oxford, England, UK; 3 Centre for Social Ethics and Policy, The University of Manchester, Manchester, UK; 4 Interdisciplinary Centre for Ethics and Institute of Philosophy, Jagiellonian University, Kraków, Poland; 5 Department of Philosophy, University of Granada, Granada, Spain

**Keywords:** Experimental philosophy, Empirical bioethics, Experimental philosophical bioethics, Normative inference, Moral judgment

## Abstract

This paper explores an emerging sub-field of both empirical bioethics and experimental philosophy, which has been called “experimental philosophical bioethics” (bioxphi). As an empirical discipline, bioxphi adopts the methods of experimental moral psychology and cognitive science; it does so to make sense of the eliciting factors and underlying cognitive processes that shape people’s moral judgments, particularly about real-world matters of bioethical concern. Yet, as a normative discipline situated within the broader field of bioethics, it also aims to contribute to substantive ethical questions about what should be done in a given context. What are some of the ways in which this aim has been pursued? In this paper, we employ a case study approach to examine and critically evaluate four strategies from the recent literature by which scholars in bioxphi have leveraged empirical data in the service of normative arguments.

## Introduction

In 2019, the first international conference on experimental philosophical bioethics (bioxphi) was held at Yale University [1]. The aim of the conference was to bring together experimental philosophers working on bioethical issues and bioethicists interested in using experimental methods from cognitive science and experimental moral psychology to inform their normative inquiry. A short position statement was drafted, and later published, spelling out the distinctive features of this emerging sub-field of both empirical bioethics and experimental philosophy (x‑phi) [2].

One important aim of the workshop and the ensuing position statement was to probe the relationship(s) between, on the one hand, empirical findings in the cognitive sciences and, on the other hand, normative or other philosophical questions at the heart of bioethics. In this paper, we aim to build on that ambition by exploring in greater detail how bioxphi studies have already offered normative insight into key bioethical issues, including the criteria for death determination and the necessary conditions for giving valid consent. Through a series of case studies, we analyze four argumentative strategies adopted more or less explicitly in recent scholarship. These case studies help to illustrate the pragmatic spirit of bioxphi, by which it seeks to build bridges between the empirical and normative programs of bioethical inquiry. At the same time, the cases represent good-faith attempts to assuage the concerns of moral philosophers who caution against drawing normative conclusions directly from empirical facts, as we will discuss.

Let us start by saying what this paper is not. It is not an attempt to recategorize existing methods or approaches within the parent disciplines of bioxphi, namely x-phi and empirical bioethics, nor does it seek to place those methods or approaches into a new typology. With respect to empirical bioethics research, for example, we recognize that a large number of methodologies have been employed which are not accounted for in the case studies below (see, e.g., [3]). Consequently, by exploring these four recent strategies for drawing normative conclusions from premises that include empirical content, we do not suggest that these are the only strategies bioxphi practitioners should employ. Rather, we hope to elucidate such strategies as they are currently being pursued within bioxphi, while remaining open to the possibility that other strategies from empirical bioethics could be adapted to research that rests more heavily on experimental study designs. To this end, we will start by situating this new area of research within its historical and disciplinary contexts.

### Situating bioxphi: principlism, empirical bioethics, and experimental philosophy

Broadly speaking, bioethics grew out of the need to make real-world moral decisions in response to gross human rights abuses, from Nazi war crimes to the Tuskegee Syphilis Study [4, 5]. Rapid innovations in health care during the 1960s also raised pressing ethical questions, including questions about how to fairly allocate life-saving technologies [6]. By the 1970s, it was clear that the field lacked a unifying theory for guiding such moral decisions, with different practitioners relying on different meta-ethical frameworks along with their own individual moral judgments about particular cases. Responding to this situation, scholars Tom Beauchamp and James Childress led the development of a shared normative framework that sought to incorporate long-standing clinical duties to maximize benefits and minimize harms, while also addressing concerns about justice and autonomy [7]. This approach was thought to provide a common source of normative guidance that bioethicists from a range of theoretical backgrounds could appeal to in their deliberations.

The four principles approach to bioethical analysis remains widespread to this day [8]. But even taking such principles for granted, questions remains about how they should be applied to specific scenarios or balanced against one another in situations where they seem to conflict [9]. A common strategy has been to take a coherence-seeking approach in which the practitioner tries to achieve a kind of reflective equilibrium. That is, she attempts to “harmonize all the elements contributing to moral judgment, including intuitions about cases, moral principles, moral theories, and background theories of moral agency and social organization” [10]. Within this method, moral theories and principles function to “organize, explain, criticize, and extend our intuitive responses to cases,” while at the same time, “those very responses can, in turn, help us to amend and sharpen our principles and theories when they prove inadequate to the complexities of emerging cases” [10].

To better appreciate this general strategy, it is necessary to ask who the implied “we” is in the reference to “our” intuitive responses to cases. For the sake of clarity, the responses of interest here are judgments regarding particular bioethical scenarios—for example, judgments that a certain course of action is morally preferable to another or that a given behavior is permissible or impermissible. These sorts of judgments have, traditionally, been those of the bioethicist, philosopher, theologian, or legal professional (in what follows we will shorten this list to “bioethicist” for simplicity). In this way, the judgments of particular individuals have long provided a key source of data for “armchair” approaches to bioethics, including the coherence-seeking kind described above. But what if the judgments of bioethicists differ from those of lay people [11, 12], or from those of other stakeholders with different levels of expertise who operate across the relevant domains (e.g., health care practitioners, policymakers, patients, their families)? One possibility is that a bioethicist’s speculative reflection, especially on abstract or idealized cases, might fail adequately to capture the concrete normative and empirical issues at stake in clinical contexts.^1^ This, in turn, might call into question the real-world relevance of such reflection for both practice and policy.^2^ If the goal is to develop a normative position regarding everyday clinical practice, for example, might the judgments of doctors or their patients constitute more relevant data? And supposing that the relevant empirical data pertaining to various stakeholder judgments have been identified, (how) can normative inferences be drawn on the basis of such data?

Empirical bioethicists have responded to these questions in a number of ways. According to a recent systematic review [3], the majority of methodologies employed in empirical bioethics can be classed as either *dialogical* or *consultative*. Dialogical approaches involve actual dialogues between researchers and stakeholders aimed at reaching a shared understanding and a joint resolution to a particular bioethical problem. Consultative approaches involve collecting empirical data relating to stakeholder views, attitudes, and experiences, and then using these as a basis for drawing normative conclusions. For the majority of consultative approaches, the end goal is the achievement of coherence either between stakeholder data and moral theory (narrow reflective equilibrium) or between stakeholder data and broader considerations, such as background theories, moral principles, expert intuitions, morally relevant facts, and considered judgments (wide reflective equilibrium).

According to Rachel Davies and colleagues, the key difference between dialogical and consultative methods is the role of participants: whereas participants in dialogical approaches work together with researchers to analyze stakeholder data and develop normative conclusions regarding discrete problems on the basis of consensus, participants in consultative approaches do not take part in the analysis or the process of forming normative conclusions [3]. Furthermore, the aims of consultative approaches vary, “ranging from theory development to the generation of concrete answers to discrete problems” [3, p. 7]. In addition, when consultative approaches aim to reach normative conclusions regarding a specific problem, they tend to employ coherence-based methodologies like the ones described above [3].^3^

Now we can ask how practitioners of x-phi, the other parent discipline of bioxphi, glean (meta)philosophical insights—including but not limited to normative inferences—from empirical data regarding human psychology. The answer to this question depends on how these practitioners interpret the purpose or function of x-phi in general. At least two main purposes have been identified, corresponding to two separate research programs, each of which can be understood in relation to the tradition of conceptual analysis in analytic philosophy [20–25].

The first program aims to make a positive contribution to conceptual analysis, though not necessarily through the provision of necessary and sufficient conditions for concept application (*positive x-phi*). The second program engages negatively by providing evidence against the intuitive assumptions of more traditional approaches to conceptual analysis (*negative x-phi*). However, as Joshua Knobe argues, regardless of which program they may claim to be pursuing, what x-phi researchers typically do in their studies is investigate experimental effects on psychological structures thought to underpin judgments held by participants [22, p. 42]. Consequently, the *cognitive science* characterization of x-phi suggests that experimental philosophers study causal effects on people’s judgments in order to map and explain such judgments using theories about underlying cognitive processes [22, p. 50]. As we demonstrate in the five case studies below, it is this characterization of x-phi as cognitive science that is especially important for understanding what some recent bioxphi studies have been trying to achieve, specifically in terms of generating normative conclusions from premises that include empirical assertions.

### Bioxphi: aims and experiments

Building on the insights and methodological approaches of its parent disciplines, bioxphi seeks to contribute to three main aims. It seeks to (1) facilitate studies of a wider range of stakeholder judgments, going beyond those of professional philosophers, bioethicists, and the like, (2) investigate how these judgments play out in more ecologically valid contexts (e.g., by employing thought experiments or examples that closely resemble the features of clinical or other real-life situations), and (3) develop a richer understanding of the underlying cognitive processes and eliciting factors that shape the judgments themselves, such as by modeling the “variation in judgments across cases as a function of carefully controlled variables” [26, p. xxiii]. It is the last of these aims, with its focus on experimental methods, that distinguishes bioxphi—methodologically—from empirical bioethics; and the second aim, with its focus on realistic bioethical scenarios, that distinguishes it from x-phi more broadly construed.

Within the four approaches to bioxphi discussed below (parsimony, debunking, triangulation, and pluralism), the running theme is that a deeper understanding of the criteria underlying judgments about bioethical cases can help to address important normative questions in bioethics. Taken together, then, we suggest that the three aims just mentioned—broadening the pool of judgments, increasing ecological validity, and unpacking causes and cognitive mechanisms—may help researchers discern whether, when, or under what conditions bioethical judgments should be accorded some degree of normative weight. Nonetheless, the experimental study of bioethical judgments alone does not exhaust the scope of this emerging field. Bioxphi can also involve experiments probing people’s use of ethical concepts or means of drawing moral inferences (among other psychological phenomena) as these relate to bioethical decision-making and policy development (see, e.g., [22–24, 26]).

Something more concrete should be said about the kinds of experiments under discussion, given that these are part of what distinguishes bioxphi from empirical bioethics. Although virtually any experimental method drawn from cognitive science or moral psychology could be adapted to a bioxphi context, the dominant method of x-phi for the past few decades, and hence of bioxphi more recently, has been a particular type of modified questionnaire involving the so-called contrastive vignette technique (CVT) [27–29]. In its simplest form, the CVT involves designing a pair of vignettes that carefully describe a particular situation but differ by a specific detail that is expected to impact on participant responses (e.g., how much they think an agent described in the vignette is morally responsible for bringing about some outcome). This detail constitutes the experimental manipulation. The study then seeks to investigate the effects of the experimental manipulation on participant responses (see, e.g., [30]).

Explicit responses, such as moral judgments, typically are measured on a Likert-type scale or continuous visual analog slider, and statistical comparisons are made between the distributions of responses provided by the two groups. Because participants are randomly assigned to one or the other group, mean differences between the resulting response distributions should be attributable to the experimental manipulation itself. This is very similar to experiments in other areas of research, such as placebo-controlled studies in medicine or crop yield prediction trials in agriculture.

In experimentally testing the factors that influence participant judgments, bioxphi shares methodological features with x-phi. The latter discipline, in turn, has borrowed methods from cognitive science and experimental psychology.^4^ However, it should be noted that although x-phi (and hence bioxphi) has tended to rely on experimental survey methods, such as the CVT, proponents have suggested that it should employ the full range of experimental methods used in the psychosocial sciences in order to expand the range of questions addressed [26, p. xxii]. This could involve, for example, the use of transcranial magnetic or direct-current brain stimulation devices to influence neural processes thought to be involved in shaping normative judgments [35]; the careful administration of psychoactive substances, such as psilocybin or MDMA, that appear capable of altering the moral sensibilities of the user [36]; or a combination of experimental and non-experimental approaches, such as interviews, qualitative studies, analyses of linguistic corpus data, and anthropological work [30, 37]. Regardless of the specific empirical methods employed, a major difference between x-phi and bioxphi is that the latter explicitly aims to inform *normative* discussions within bioethics.

## Normative inference: from premises with empirical content to normative conclusions

Recall that bioxphi seeks to study the moral judgments of a broad range of relevant stakeholders; that it seeks to do so in an ecologically valid manner that captures realistic pertinent features of bioethical situations; and that it uses experimental methods to identify the elicitors and psychological processes that yield these judgments. To what extent, and in what ways, should empirical findings regarding stakeholder judgments play a role in devising solutions to bioethical problems?

Let us start by dealing with a red herring. We take it as uncontroversial that the most ethically justified conclusion is not always, or simply, the most popular one based on common opinion. However, as we acknowledge in the next section, empirical approaches to bioethics can, when certain conditions are met, appeal to the presence of prevalent, robust, or highly consistent stakeholder judgments revealed by a study as one (ultimately defeasible) reason that counts in favor of a particular normative claim.

At the same time, we recognize that simply deferring to putative ethical experts can also be problematic, especially when their judgments or associated normative conclusions defy those of other stakeholders. Such knee-jerk deferral to a circumscribed group of people can enshrine prejudices and lead to dogmatism and parochialism [23, pp. 126–148]. As Julian Savulescu and colleagues observe, some laws and policies do in fact run counter to public preferences [38]: bans on voluntary assisted dying in the United States, United Kingdom, and Australia, for instance, have been put in place despite large majority preferences for permitting assisted dying [17–19]. Other laws and policies, however, seem to be grounded in mere public sentiment, which is to say they reflect majority attitudes—including attitudes of repugnance toward newfangled technologies—without necessarily appealing to more principled normative considerations [8, 15–19].

Typically, neither a direct appeal to an *argumentum ad populum* nor a simple appeal to a single set of supposedly expert judgments will provide reliable guidance toward normative solutions, proposals, or arguments that meet the needs of patients, practitioners, and policymakers. To get out of this bind, Savulescu and colleagues have introduced a number of preliminary proposals. First, they suggest that it is necessary to identify the intuitions of those who have been careful in their reasoning and have a “clear understanding of the issues” [38]. But who are these careful and reliable reasoners and how does one find them? They might be among those ethical experts previously mentioned: professional moral philosophers, bioethicists, legal professionals, and the like. However, members of these groups make up a tiny fraction of the population, and between them they may have idiosyncratic perspectives, conflicting judgments, moral disagreements, and incompatible moral inferences.

Furthermore, debates surrounding a specific bioethical or philosophical issue may have reached a stalemate with good reasons for adopting several positions and/or no adequate way for the experts to agree upon which position should be implemented in policy and practice. Consequently, Savulescu et al. offer the view that ethical policy should rely not only on “refined expert intuitions,” along with guidance from formal ethical theories, but also on “widespread public responses” [38].

A potential problem with such proposals is that they do not spell out what to do when widespread public responses, such as common moral judgments, and those of putative experts diverge or, indeed, what to do when they seem to agree. More generally, how do ethical theories, expert judgments, and the judgments of ordinary people relate to one another, and how can this information be integrated to draw normative conclusions? We will employ a series of bioxphi case studies to articulate and explain the four integrative approaches mentioned above: *parsimony*, *debunking*, *triangulation*, and *pluralism*.

We do not conceive of these approaches as (necessarily) fitting into an overarching step-wise procedure. That is, we do not suggest that one should start with parsimony, move to debunking, then to triangulation and end with pluralism. Rather, answering a bioethical question in the field of bioxphi might involve the application of just one of these suggested approaches, or more than one, either in parallel or in varying sequence. Indeed, as we noted earlier, bioxphi studies might conceivably use still other methods not covered here. Accordingly, our aim is modest: it is to shed light on a small selection of recent strategies in the burgeoning field of bioxphi for generating normative conclusions from premises that include empirical components.

## The parsimony approach

The first strategy is based on a principle of parsimony. This view assumes that ordinary people’s judgments about certain cases carry significant (albeit defeasible) normative weight, such that experts who wish to make claims about what ought to be done should begin by carefully studying those judgments. The strategy is parsimonious, then, in that it relies on the simplest possible model for deriving normative content from the moral judgments of ordinary people: it holds that those judgments should be given at least some positive normative weight. In short:

**Parsimony.** If relevant stakeholders consistently make a judgment *p* which encodes moral claim M, then M has prima facie normative weight.

One of the aims of studies employing this strategy is to gather data relating to stakeholder judgments, often with the assumption that no matter what these judgments are, they are normatively significant. Once the data have been gathered, a proponent of the parsimony strategy might then identity the most consistent (e.g., common or robust) moral judgments revealed by a study and give these prima facie normative weight when deciding on a solution to an associated bioethical problem. Note that the normative weight accorded to such judgments need not be especially strong. The parsimony strategy requires only that these judgments, to the extent that they are consistent or prevalent, be viewed as providing *some* normative weight in a moral-philosophical argument. They will never be enough on their own to deliver an all-things-considered normative conclusion.

In this way, bioxphi studies that employ the parsimony strategy are consistent with consultative approaches to empirical bioethics, insofar as the latter rely on the robust judgments of some group of stakeholders as a basis for arbitrating between competing normative claims. However, consultative approaches in empirical bioethics tend to be concerned with identifying the most prevalent judgments (or attitudes, preferences, etc.) of the group, primarily through observational or cross-sectional methods. By contrast, bioxphi studies that adopt a parsimony strategy typically look beyond the mere prevalence of a given judgment and instead emphasize the robustness of an experimental finding (e.g., across methods, materials, or operationalizations of a causal stimulus) regarding a given effect on participant responses (see, e.g., [39]).

There are two interrelated concerns with employing this approach in the context of bioxphi studies. First, it is highly likely that traditional moral philosophers would hesitate to accept such a method on the basis that it seems to derive normative conclusions from empirical premises without necessarily appealing to more principled normative considerations. Therefore, although the parsimony approach has been employed in the field of bioxphi, as seen in case study 1, alongside broadly similar consultative approaches in the domain of health-related public policy [8, 15–19], we must reiterate that it is not an effective means of delivering all-things-considered normative solutions to bioethical puzzles.

This caveat relates to a second potential concern, which is that the parsimony approach seems to reduce bioethics to a popularity contest. We think this concern is unwarranted. As noted earlier, the fact that a study reveals consistent normative judgments does not entail that the associated bioethical issues are thereby conclusively settled. Rather, consistent judgments are just one factor counting in favor of a given moral claim, and the normative weight accorded need not be strong. Indeed, reasons for granting more normative weight to a particular set of judgments are, ultimately, defeasible if it can be convincingly shown that these judgments are unreliable or outweighed by some other moral consideration. In short, this approach puts the burden of proof on those who would claim that one should *not* respond to the ethical issue in question by according some normative weight to the most consistent judgments of relevant stakeholders.

**Table:** 

**Case study 1.** Parsimony
“Judgments of moral responsibility in tissue donation cases” [40]
Consider a child who needs a tissue donation in order to survive. Suppose that her biological parent could donate the needed tissue. Insofar as it seems intuitive that the parent has a moral responsibility to donate the tissue, what drives this judgment? Is it the biological relation between the donor and recipient [41] or the fact that the donor is uniquely suited to provide tissue that will work for the recipient [42]? John Beverley and James Beebe, in a study involving a series of contrastive vignettes, found that “unique ability rather than biological relatedness was the primary predictor of people’s judgments of moral responsibility” [40]. To distill the normative relevance of this finding, the authors adopt a metaphilosophical stance: folk judgments need not “rigidly constrain philosophical theorizing,” but counterintuitive normative views (e.g., that moral responsibility stems from biological relatedness) carry an explanatory burden [40]. As such, the parsimony model would maintain that the “unique ability” judgment be assigned prima facie normative import.

## The debunking approach

In contrast to the parsimony approach, which assigns prima facie (though not necessarily strong) normative weight to stakeholder judgments, one might wish to argue that certain judgments should *not* be accorded normative weight when considering a solution to a given bioethical problem. One might make such an argument by testing whether the judgment is unreliable, in the sense that the psychological processes outputting the judgment are not disposed to produce a sufficiently large proportion of true judgments [23, p. 96]. In other words, a judgment is unreliable if it is the result of a psychological process that is not disposed to reliably either “arrive at the truth” [43, p. 227] or “track the truth” [44, p. 54] as measured by some agreed-upon standard. A judgment could be unreliable in this way if it is the output of a psychological process that, for example, has been substantially influenced by prejudice, epistemological distortions, or morally irrelevant differences in how a case is presented (i.e., framing effects). In order to investigate whether a judgment should *not* be accorded normative weight in a bioethical argument, one might wish to pursue a debunking strategy derived from the following general argumentation scheme employed in x-phi (adapted from [24, pp. 31–56]):

**Debunking.**

1. Judgment *p* is the output of a psychological process that possesses the empirical property of being substantially influenced by factor F. (empirical premise)
2. If a judgment is the output of a psychological process that possesses the empirical property of being substantially influenced by factor F, then it is pro tanto unreliable. (bridging normative premise)
3. Judgment *p* is pro tanto unreliable.

Such an approach can be employed to assess whether ordinary people revise their judgments under various treatment conditions. Take exposure to a particular philosophical argument. If people update a given judgment about a specific bioethical case after having reflected on a philosophical argument, then at least two points might follow: (1) they had not previously considered the philosophical argument in question; and (2) once they did, they abandoned their original judgment. But even if a particular judgment is shown to be pro tanto unreliable, why is this a prima facie reason for others to disbelieve the judgment?

The literature on moral judgments and their susceptibility to framing effects delivers two interrelated answers. First, as James Andow observes, the substantive influence of morally irrelevant factors on judgments is important because “it is capable of radically altering the moral position that one ends up endorsing” [45, p. 908]. If a person’s judgment about some case is the output of a psychological process that has been substantively influenced by a morally irrelevant factor, then there is a prima facie reason to doubt that judgment. But more than this, there is a reason to believe that a process of reflection based on that judgment “will only lead one deeper into error” [43, p. 244]. At least, the pro tanto unreliability of a judgment is one factor that counts against accepting it as a premise in a normative argument.

Second, according to Edouard Machery, “when a judgment-forming process is unreliable, the judgments it produces are severely deficient from an epistemic point of view” [23, p. 99]. Accordingly, the unreliability of a judgment undermines its justificatory status; that is, the judgment is not justified without independent confirmation that grants the judgment holder the ability to infer that judgment [44]. Here, when we say that a judgment is justified, we mean that a person ought to make it as opposed to suspending it, because she has “adequate epistemic grounds for believing that it is true (at least in some minimal sense)” [44, p. 48].

Accordingly, if a bioxphi study finds that a judgment is unreliable to the extent that it is the output of a psychological process that is subject to the substantive influence of a morally irrelevant factor, then there are inadequate epistemic grounds for believing that it is true—unless an individual can independently confirm the unreliable judgment by making some appropriate inference or argument [44, p. 51]. It follows that its unreliability is a prima facie reason for a person to suspend her judgment in relation to the bioethical case in question. Consequently, through the debunking approach, the result of empirical investigation—namely, the pro tanto unreliability of a judgment—can be used as a prima facie reason that counts against the normative weight of the judgment in an argument that might be developed on the basis of it.

But a word of caution is warranted. When bioxphi studies adopt a debunking strategy, it should not be assumed that those conducting the study believe that the target judgment should in fact be accorded normative weight in a given argument. Rather, prior to pursuing the debunking approach, researchers in bioxphi would already have identified a normative argument or conclusion (for example, in the published literature) that appeals to this target judgment. The purpose of the bioxphi study in this context would be to employ the debunking approach to demonstrate that the judgment in question is, or is not, pro tanto unreliable. Evidence that it is in fact unreliable could then be interpreted as debunking the pre-existing normative argument.

In addition, when stakeholders hold a judgment that has been shown to be pro tanto unreliable in a specific instance, alternative explanations should be explored for why the target population holds that judgment. After all, one can never be sure that a particular judgment can be debunked in general: debunking proceeds by looking at isolated, specific ways in which the psychological processes outputting certain judgments can be deficient (see footnote 5). To see what we mean, consider a hypothetical study, inspired by Edmond Awad et al. [46], in which participants tended to favor saving females over males in a life-threatening situation, all else being equal. According to most contemporary moral theories, a sex-based distinction by itself is not a valid basis for treating otherwise equal persons differently. In such a case, however, the revealed judgment favoring females over males could turn out to be a mere proxy for some other distinction that is, in fact, widely agreed to be normatively relevant. Suppose that males vastly outnumber females in the surveyed society, leading to significantly negative social consequences. If participants from that society make a judgment that females should be saved over males, then it might not be sex per se that is driving their survey responses, but rather a preference for rebalancing a skewed sex ratio and/or alleviating attendant social ills.

However, even when remaining considerate of possible alternative explanations, one must also acknowledge that judgments can be affected by a number of factors, and at least some will be irrelevant to the moral question. Whether a particular pro tanto unreliable judgment is deemed to be relevant will, as in this example, depend on background theoretical assumptions that independently confirm the judgment, grounding the inference of those who infer it [44]. In this case, the relevance of the judgment that females should be saved over males depends on the principled normative basis by which sex-based judgments are deemed justifiable under one set of conditions but not another.

Experimental philosophical bioethicists can play a valuable role in cases where there are plausible alternative explanations for what appears to be a normatively unjustified judgment. In particular, they can conduct experiments to test the alternative explanations, often by carefully manipulating relevant contributing factors. For instance, in the above study, researchers might observe whether a pro tanto unreliable judgment persists when participants are asked to consider a hypothetical society in which females outnumber males. Depending on the results, such an experiment could provide evidence for or against the alternative explanation for the original suspect judgment. In what follows, we show how the debunking approach might be pursued in practice, using two separate case studies drawn from the burgeoning bioxphi literature: (1) case study 2 highlights an apparently failed debunking attempt, providing evidence that ordinary people’s judgments, in a specific instance, are not largely biased by a particular factor that would have made the judgments unreliable; and (2) case study 3 highlights an apparently successful debunking attempt, providing evidence that ordinary people’s judgments, in a specific instance, are largely biased by a particular irrelevant factor and so should not be trusted in the relevant case.^5^

**Table:** 

**Case study 2.** Debunking: failed
“How do people use ‘killing’, ‘letting die’, and related bioethical concepts?” [49]
Laypeople distinguish killing and letting die by evaluating the morality of the physician’s intervention [50]. For example, doctors who employ end-of-life interventions that honor the wishes of terminal patients are seen as allowing them to die, whereas doctors who employ interventions that disregard patients’ wishes are seen as killing them [49]. The judgments of ordinary people may afford little normative insight here, in part because they lack the requisite understanding of the medical and clinical issues in play. This objection makes a straightforward empirical prediction: if laypeople acquired the relevant medical knowledge, they would abandon their untrained judgments in favor of the canonical distinction between killing and letting die as commissive versus omissive life-ending acts, respectively. However, David Rodríguez-Arias and colleagues found no evidence of this: doctors, medical students, and laypeople revealed strikingly similar judgments about end-of-life cases [49]. The determining factor appears to be whether the patient wished to live or die, and not how the patient’s death was brought about (i.e., via action or omission). Thus, the ordinary judgment could not be debunked on grounds of ignorance of clinically relevant details.

**Table:** 

**Case study 3.** Debunking: successful
“Gender bias in pediatric pain assessment” [51]
Do people have a gender bias in assessing children’s pain? To answer this question, Brian D. Earp and colleagues conducted an experiment in which they manipulated the perceived gender of a young child getting a finger-stick to draw blood (based on [52]). To keep the experiment as controlled as possible, participants viewed a single video stimulus of a child whose sex could not be visually determined (i.e., the same video in both conditions). In one condition, participants were told the child’s name was Samuel, and in the other, Samantha. Participants then watched the video and rated how much pain the child experienced. Earp et al. found that participants rated the child named Samuel as experiencing more pain than the child named Samantha. Thus, perceived gender alone appeared to bias observer interpretations of felt pain (for alternative explanations, see [53]). Such evidence plausibly undermines the trustworthiness of judgments that, say, boys and girls should receive different pain treatment given a comparable injury.

## The triangulation approach

Suppose that the judgments of philosophers, bioethicists, legal professionals, or those with other kinds of expertise differ from the judgments of lay people. Alternatively, suppose that there are conflicting judgments within the domain of expert stakeholders, such as between bioethicists, legal professionals, health care practitioners, policymakers, and patients. What should be done about such divergences? In such cases, experimental philosophical bioethicists could helpfully pursue a coherence-seeking strategy of narrow reflective equilibrium discussed previously. Accordingly, the coherence they seek will be between competing expert judgments and/or between expert judgments and those of lay stakeholders. We refer to this approach as a type of triangulation.^6^

**Triangulation.** Divergence among the judgments of various groups of experts and/or between expert and lay judgments requires the following: adjusting, pruning, or supplementing the normative conclusions derived from either expert or lay judgments in order to accommodate the normative implications of the opposing views.

Experimental philosophical bioethicists can perform three important roles in pursuing a triangulation strategy. First, using empirical means, they can identify the judgments of various experts and lay stakeholders in response to a specific normative problem, ensuring that the judgments respond to relevant features of ecologically valid contexts. Second, using the aforementioned argumentation strategies of x-phi and the methods of cognitive science and experimental psychology, they can experimentally investigate the cognitive mechanisms underpinning these judgments, ensuring that various expert and lay judgments are not pro tanto unreliable (and/or setting aside or discounting those judgments that are shown to be pro tanto unreliable). Finally, they can execute trade-offs among the respective pro tanto reliable judgments, revising normative conclusions as coherence and mutual support seem to require.

According to the standards of reflective equilibrium, the normative conclusions arrived at through this process, together with revisions to competing judgments, will be justified if and only if there is reason to believe that they will maximize the coherence of the overall set of relevant considerations. However, in order to avoid the standard objection that the equilibrium arrived at “may be no more than a reshuffling of moral prejudices” [55, p. 22], the triangulation approach might be better characterized as a coherence-seeking methodology based on a “moderate foundationalism” [56, pp. 26–30]. The problem that Richard Brandt identifies is that the coherence constraint on its own may not succeed in correcting for all the errors or biases in the respective judgments [55]. As already observed in the section on debunking, it will not succeed if the antecedent judgments are so unreliable that further reflection on these judgments will only lead bioethicists deeper into error. As a result, proponents must also explain how it is antecedently or independently rational to regard some or all of these competing judgments as (pro tanto) reliable [43].

It is at this point that we can appreciate one of the opportunities afforded by bioxphi: by employing experimentally based argumentation strategies that, in principle, give reasons to believe that certain judgments are not the output of psychological processes that have been substantively influenced by (particular) irrelevant factors, bioxphi allows conclusions to be drawn regarding the pro tanto reliability of those judgments. Once certain judgments have survived various attempts at being shown to be pro tanto unreliable,^7^ the resulting judgments can be employed as initially credible judgments in the given process of triangulation. Such an approach is moderately or modestly foundationalist because the degree of justification provided by the expert or stakeholder judgments that are each accepted as pro tanto reliable may be relatively low. Proponents of reflective equilibrium maintain that sufficient justification requires mutual support among the set of judgments—and, in many cases, among judgments, background theories, principles, and morally relevant facts [57].

According to T.M. Scanlon, those adopting the model of reflective equilibrium should ask whether there is more reason to revise a normative conclusion in the light of conflicting judgments or to give up the judgments that conflict with it [58]. Ultimately, as Ralph Wedgwood suggests, what is being proposed is a thoroughgoing form of fallibilism [43, pp. 64–65]. On this approach, it can never be guaranteed that one will not be rationally required to revisit and reconsider, and perhaps revise, the normative conclusions derived from the process of triangulation if and when further reliable empirical evidence is identified. In this way, reflective equilibrium, even of the narrow variety, is an ideal that is unlikely ever to be reached [43, p. 265].

In practical circumstances, when, in pursuit of “good bioethics” [8], one is required to generate arguments that meet the needs of patients, practitioners, and policymakers, one will often have to bite the bullet, so to speak, and commit to a specific normative claim on the strength of available empirical data and the degree of coherence one has been able to achieve. As Scanlon suggests, such a commitment must be based on the best reasons counting in favor of the specific claim [58].

Case study 4 provides an example of the problems that experimental philosophical bioethicists may encounter when trying to navigate between expert and lay judgments. In such cases, proceeding with a triangulation strategy may not be as straightforward as seeking a simple compromise or accommodation. Rather, it will often be necessary to ask, in the case of judgments about a normative concept, for example, what the concept is trying to *do*—that is, what its function is (as opposed to its meaning) [13, 59]. As Sally Haslanger puts it, “What is the point of having these concepts? What cognitive or practical task do they (or should they) enable us to accomplish? Are they effective tools to accomplish our (legitimate) purposes; if not, what concepts would serve these purposes better?” [60, p. 33].^8^

Once again, it is possible to envisage a vital role for bioxphi in answering such questions. As Jennifer Nado argues, “the experimental philosopher’s focus on underlying psychological mechanisms seems to be a promising route (though of course not the only possible route) for discovering the purposes our concepts serve, and the means by which these purposes are achieved” [37, p. 92]. On this view, judgments about some normative concept should be empirically investigated to determine whether the normative concept in question is already fulfilling its intended functions to a reasonably good degree.

**Table:** 

**Case study 4.** Triangulation
“Commonsense consent” [63]
In a series of experiments, Roseanna Sommers found that lay participants tended to think that deceived individuals could grant meaningful consent [63]. By contrast, in various legally relevant domains, including medicine, agreement or assent under deception is not considered valid. In this way, commonsense consent seems to diverge significantly from the notions of consent that prevail in the law and in the relevant philosophical literature. One reason this divergence matters is that lay people sit on juries that can be asked to establish whether consent was present—often without explicit guidance on how to understand consent. This empirical finding can motivate: (a) contextual education for jury members; (b) reform of the legal concept; or (c) some adjustment of both concepts in light of additional considerations (triangulation).

Depending on how the function of a bioethical concept is understood in the relevant contexts, different conclusions will be reached about how to navigate divergences among different expert judgments or between expert and lay judgments. Faced with a divergence, one might be tempted to make improvements to a given concept—that is, to increase clarity, reduce vagueness, remedy confusion, or bring about (other) desirable outcomes. Take the concept of *futility* as discussed by John McMillan [8, pp. 154–155]. On the one hand, physicians deem medical treatment to be futile if it is very unlikely to improve the well-being of the patient. On the other hand, physicians also employ the concept of futility when an intervention does not succeed as a treatment, regardless of their beliefs about the patient’s well-being. McMillan proposes to resolve the confusion between these two concepts of medical futility on the basis of their respective functions and uses.

With regard to case study 4, it might be determined that standard ethical and legal accounts of consent already adequately serve the forms of inquiry in which the concept is employed. On that basis, given that patients, physicians, and the public will be required to use the concept in legal and/or medical situations, some form of contextual reeducation might be needed to clarify, or remedy confusions in, lay judgments regarding consent—that is, to bring the lay use of the concept into line with the expert view. For instance, one application of the triangulation strategy could be to make improvements or modifications to the legal system in which the concept is employed (e.g., providing jury members with explicit definitions of the legal concept of consent and its relationship with deception) so that the intended normative work performed by the concept is no longer threatened by the divergence between expert and lay judgments.

Suppose, however, that a particular canonical concept is already clear, sharp, and free of confusion. Its application may nonetheless engender moral obligations that conflict with those that lay people intuitively recognize and, indeed, with those recognized by various other experts. In these circumstances, we might recommend wholesale replacement of a specific expert concept with an ameliorative one—namely, one that possesses a superior practical function for the forms of inquiry in which lay people and other relevant experts employ the concept. For example, in light of persistent philosophical debates (and disagreements) about the distinction between the concepts of active and passive euthanasia, new concepts, with contrasting functions, were developed: physician-assisted suicide, palliative sedation, and withdrawing life-sustaining treatment [13]. Compared with the concepts of active and passive euthanasia, these new concepts have allowed a broader range of experts as well as the public to navigate the ethico-legal terrain more successfully [13, pp. 34–35].

Nonetheless, the real problem hinges on whether the bioethical terms that have been developed for use in legal and philosophical practice are suitable for fulfilling the purposes that previous terms have served in health care and medical law contexts. However, we recognize that for conceptual reform to be based on an all-things-considered judgment, merely appealing to a divergence between expert and lay judgments will be inadequate. Although the triangulation approach is a useful starting point for conceptual reform insofar as it attempts to navigate between expert and lay judgments, it will require supplementation by, for example, seeking coherence between the diverging judgments and broader normative considerations, such as background theories, principles, morally relevant facts, and the like (wide reflective equilibrium).

## The pluralism approach

This brings us to the fourth strategy. Suppose that the best reasons count in favor of preserving two or more diverging expert judgments or the competing judgments of experts and lay stakeholders. In other words, suppose there are equally good reasons for adopting two or more different expert judgments or for adopting both an expert judgment and a lay judgment as the basis for (competing) normative conclusions, with no better reasons for adjusting, pruning, or supplementing the different positions. In some cases, such a scenario might justify a pluralistic response.

**Pluralism.** In cases where expert and lay stakeholders hold conflicting, yet pro tanto reliable, judgments or where multiple and independent communities each reveal persistent disagreement between two or more conflicting, yet pro tanto reliable, judgments, these judgments may all have comparable normative weight.

The pluralism approach is similar to, and consistent with, the shared decision-making approach that has recently become an important part of clinical practice and health policy. To be successful, shared decision-making relies on two sources of expertise: (1) the health professional as an expert on the effectiveness, probable benefits, and potential harms of different treatment options; and (2) the patient as an expert on herself, her preferences, social and personal circumstances, attitudes toward illness and risk, tolerance for pain and discomfort, long-term outlooks, and broader values [64–66]. As Jonathan Lewis notes, shared decision-making is most appropriately applied under conditions of uncertainty, which arise when a treatment decision is preference-sensitive—that is, when medical evidence and clinical expertise suggest that there is more than one medically reasonable option, and the choice of which option is best for a given patient depends on her values and preferences [66]. In short, so long as the patient can fulfill certain conditions of autonomy, then she should choose the particular intervention that best satisfies her attitudes and preferences [66–68]. Case study 5 demonstrates how a bioxphi pluralism strategy can generate sets of pro tanto reliable judgments with comparable normative weight.

**Table:** 

**Case study 5.** Pluralism
“Minds, brains, and hearts: an empirical study on pluralism concerning death determination” [69]
Pluralism concerning death determination states that people should be allowed to choose—within reason—what criterion will be applied to determine their own deaths [70]. It assumes that death determinations take place under conditions of uncertainty in the presence of more than one medically reasonable option. Against this backdrop, Ivars Neiders and Vilius Dranseika presented study participants with a possible description of the process of dying that was divided into a number of stages, beginning with the onset of health deterioration and concluding with the onset of corpse decay [69]. They were asked at which of these stages they would prefer their own death to be declared. Three of these stages were designed to mimic the main conceptions of death discussed in the bioethics literature: higher-brain death, whole-brain death, and cardiopulmonary death. Given that the data reveals widely differing preferences concerning death determination criteria, Neiders and Dranseika argue that the pluralist solution fits best with the way lay people think about death determination. If one agrees that people should be allowed to choose their own conception of death, then, given that most participants chose one of the three criteria discussed in the bioethical literature, the study provides some preliminary empirical evidence for which conceptions of death should be used in generating a choice set.

## Conclusion

Bioxphi differs in important ways from its parent disciplines. In contrast to empirical bioethics, bioxphi typically favors experimental methods, derived from cognitive science or x-phi, which help to characterize the features that shape our judgments (i.e., their determinants), as well as the processes that support them (i.e., the cognitive mechanisms). Moreover, whereas x-phi attempts to derive (meta)philosophical conclusions on the basis of empirical data, bioxphi is specifically concerned with understanding the normative implications of such data as they pertain to matters of bioethical concern.

Having traced the contours of this emerging discipline, we illustrated four ways in which experimental philosophical bioethicists have employed experimental methods to confront various normative questions that have captured bioethicists’ attention. Furthermore, we attempted to formalize the specific argumentative strategies of these approaches, while highlighting certain limitations and possible concerns inherent in each. Thus, our paper is not intended as a definitive map of the pathways leading to normativity, nor as a comprehensive defense of any single approach against the range of philosophical objections. It is more of a sampling platter, an exploration, an invitation for further discussion.

Most of the time, as the five case studies make clear, it is not obvious whether a particular scientific portrait of judgments vindicates or undermines their normative worth. Many of the case studies we presented involve ambiguous circumstances. How, then, can normative guidance be drawn from empirical research? The four approaches detailed here each provides the same answer: by bringing in further reliable evidence. Once that evidence is established, bioxphi offers an opportunity to better navigate competing views of different experts, as well as competing views of experts and lay stakeholders. Adopting a minimally realist perspective on morality, we might hope that this back-and-forth between different views (guided by theoretical frameworks and arguments) and the empirical evidence pertaining to diverging judgments can bring us closer to discerning what we have most reason to believe is morally true in the bioethical domain.

## Footnotes

1 For a robust account of the different ways in which bioethics can bring moral reason to bear on practical issues, see [8].

2 For example, see [8, 13] for potential problems with a well-known theoretical critique of the distinction between active and passive euthanasia [14].

3 Many moral philosophers hold that one cannot directly infer an “ought” from an “is.” By contrast, within the domain of health-related public policy, empirical research has often been viewed as affording direct normative insight into what should be done or recommended. For instance, the United Kingdom’s Human Fertilisation and Embryology Authority (HFEA) seems to have grounded a public policy position denying sex selection for social (non-medical) reasons on the normative weight of public opinion alone [8, 15–19].

4 Concerns have been raised about the replicability of results in experimental (social) psychology [31–33]. Meanwhile, there is evidence that x-phi studies have a replication rate of over 70% [34], more than twice as high as typical estimates generated for social psychology.

5 There is an asymmetry between these two cases. If there really is conclusive evidence that a popular judgment is grounded in some factor that undermines its normative force (successful debunking attempt), then the judgment should be set aside or discounted. But if one fails to debunk a given judgment, this does not automatically entail that it should be trusted. Rather, it might still be normatively suspect on other grounds that have not yet been tested. So one should try to test, and rule out, the most plausible debunking explanations, and if one reliably fails in this, it becomes reasonable to treat the judgment as carrying prima facie normative weight—a process akin to Popperian falsificationism, notwithstanding its various shortcomings (see [31, 47, 48]).

6 Scientists and certain philosophers of science employ the term “triangulation” to refer to the use of multiple and independent sources of evidence in order to generate causal inferences from data to phenomena (as opposed to phenomena-to-theory deductive inferences) [54]. Analogously, triangulation in bioxphi is one of the means by which experimental philosophical bioethicists have generated *normative* inferences on the basis of multiple, independent, and pro tanto reliable pieces of empirical evidence.

7 See footnote 5 for a discussion of some of the problems with attempting to demonstrate the *general* reliability of moral judgments.

8 For other broadly functionalist, experimentally based approaches to navigating conceptual disagreement, see [21, 23, 37, 61, 62].
